# Genome-wide identification of conserved intronic non-coding sequences using a Bayesian segmentation approach

**DOI:** 10.1186/s12864-017-3645-2

**Published:** 2017-03-27

**Authors:** Manjula Algama, Edward Tasker, Caitlin Williams, Adam C. Parslow, Robert J. Bryson-Richardson, Jonathan M. Keith

**Affiliations:** 1 School of Mathematical Sciences, Monash University, Melbourne, VIC 3800 Australia; 2 School of Biological Sciences, Monash University, Melbourne, VIC 3800 Australia

**Keywords:** ncRNA, Conserved non-coding sequences, Putative functional elements, Genome segmentation, Bayesian modelling

## Abstract

**Background:**

Computational identification of non-coding RNAs (ncRNAs) is a challenging problem. We describe a genome-wide analysis using Bayesian segmentation to identify intronic elements highly conserved between three evolutionarily distant vertebrate species: human, mouse and zebrafish. We investigate the extent to which these elements include ncRNAs (or conserved domains of ncRNAs) and regulatory sequences.

**Results:**

We identified 655 deeply conserved intronic sequences in a genome-wide analysis. We also performed a pathway-focussed analysis on genes involved in muscle development, detecting 27 intronic elements, of which 22 were not detected in the genome-wide analysis. At least 87% of the genome-wide and 70% of the pathway-focussed elements have existing annotations indicative of conserved RNA secondary structure. The expression of 26 of the pathway-focused elements was examined using RT-PCR, providing confirmation that they include expressed ncRNAs. Consistent with previous studies, these elements are significantly over-represented in the introns of transcription factors.

**Conclusions:**

This study demonstrates a novel, highly effective, Bayesian approach to identifying conserved non-coding sequences. Our results complement previous findings that these sequences are enriched in transcription factors. However, in contrast to previous studies which suggest the majority of conserved sequences are regulatory factor binding sites, the majority of conserved sequences identified using our approach contain evidence of conserved RNA secondary structures, and our laboratory results suggest most are expressed.

Functional roles at DNA and RNA levels are not mutually exclusive, and many of our elements possess evidence of both. Moreover, ncRNAs play roles in transcriptional and post-transcriptional regulation, and this may contribute to the over-representation of these elements in introns of transcription factors. We attribute the higher sensitivity of the pathway-focussed analysis compared to the genome-wide analysis to improved alignment quality, suggesting that enhanced genomic alignments may reveal many more conserved intronic sequences.

**Electronic supplementary material:**

The online version of this article (doi:10.1186/s12864-017-3645-2) contains supplementary material, which is available to authorized users.

## Background

Functional, non-coding, genomic sequences carry out important cellular functions. These sequences can include enhancers and silencers, regulating gene expression, and non-coding RNAs (ncRNAs). ncRNAs have been implicated in a variety of biological functions including chromatin modification [1–3], transcription [4], and RNA splicing [5, 6], editing [7], and translation [8]. Despite the increasing evidence of their importance the tools available for the detection of functional non-coding elements in a genome, in contrast to the array of tools available to identify coding sequences, are limited. This is largely due to the wide range of non-coding elements and the lack of characteristic features to assist in their identification.

Current computational methods to identify ncRNAs; such as Mfold [9], RNAfold [10], and RNAz [11], rely on formation of secondary structure, or combine this approach with comparative sequence analysis (such as EvoFold [12]). The formation of secondary structures is a feature of many ncRNAs including; small nucleolar RNAs, tRNAs, and microRNAs; but many ncRNAs and non-coding regulatory sequences will be missed using this approach.

Conservation of sequence between species is widely used as an indicator of function. Conservation can be identified using a sliding window analysis applied to whole-genome alignments. This technique involves counting the number of matches/mismatches in overlapping windows of a predetermined length, to obtain a profile of conservation level across the sequence. Many previous studies have used such analyses to identify conserved non-coding sequences in human and other genomes [13–17]. Two key findings have emerged from these studies. Firstly, there is strong evidence, both computational and experimental, that conserved non-coding sequences are highly enriched in regulatory sequences, especially regulatory element binding sites [13, 18–20]. A second finding is that conserved non-coding sequence is selectively located near transcription factors and genes involved in development and the nervous system [15–17, 20, 21].

Sliding window analyses have several limitations. A smaller window allows for more precise localisation of changes in the property of interest but also allows for noise within the sequence to more significantly affect the output. Thus sliding window analysis is inherently a compromise between these two factors [22]. The technique also fails to precisely localise boundaries in functional elements, such as the boundaries between exons and introns, the ends of transcription factor binding sites (TFBSs), and the transcription start sites of expressed RNAs, for which more sophisticated segmentation methods are required [23, 24]. The second disadvantage is the common consideration of conservation as a dichotomy (conserved or not-conserved), whereas in reality the constraints on any given region will differ resulting in multiple classes of conservation within a genome. For example, analysis of genome alignments from drosopholids and mammals identified 7 and 9 evolutionary rate classes respectively [25]. As a result it is not possible to set threshold values for conserved elements that will consistently identify non-coding functional elements.

To overcome the above-mentioned disadvantages we performed an analysis using changept, a Bayesian segmentation model [26, 27]. Adopting a Bayesian approach is beneficial as it provides quantification of the uncertainties in parameter estimates in the form of probability distributions. The changept model can be described as a segmentation-classification model, which is capable of simultaneously segmenting a genomic alignment and classifying segments into one of a predefined number of segment classes. Segments are classified according to multiple sequence characteristics including level of evolutionary conservation between species, GC content and transition/transversion ratio, and precise boundaries for the segments are identified.

Using changept, we carried out a genome-wide analysis using an automated alignment of the zebrafish, mouse, and human genomes. It is possible to apply changept to an alignment of a large number of species, using one of the alignment encodings introduced in [25]. However, these encodings focus on the conservation properties of the alignment only. Alignments contain additional information indicative of function, including variations in GC content and in transition/transversion ratio. Here we consider an alignment of only three species, so that we can use encodings that capture this additional information [28]. We chose zebrafish and mouse genomes as these are potentially useful model organisms for future investigations of functional significance.

We identified 655 intronic putative functional elements (PFEs) distributed among 193 zebrafish genes and compared these to predictions from other approaches and to sequence databases. Using analysis of sequence conservation we identified many elements that had previously been identified using secondary structure analysis, and some novel elements. We also identified that the PFEs were highly enriched in transcription factors. To examine if there were conserved elements between different members of the same pathway and the effects of optimised local alignments, we performed a pathway-focussed analysis on 24 genes involved in muscle development, identifying a similar enrichment in transcription factors and that conservation rates not only vary across the genome but also within a single gene. We identified 27 PFEs in genes in the myogenesis pathway that belong to the class of most highly conserved segments. We validated our findings experimentally, confirming the expression of these intronic elements in zebrafish embryos.

## Results

To identify putative functional non-coding elements conserved between human, mouse and zebrafish, we performed a genome-wide analysis using the readily available multiz 8-way alignment. For each zebrafish chromosome, a zebrafish-referenced 3-way alignment was extracted, giving 25 alignments in total. Approximately 4–5% of each chromosome was aligned, however this captured 50% of the Ensembl genes.

### Identification of conserved non-coding elements

To search for the most conserved elements in each gene, changept was applied to each chromosome alignment independently. Alignments were segmented into classes, based on conservation rates, and with the number of classes determined set to be the minimum number that could be fitted to the data. For each class the posterior probability that each sequence position belongs to the class was determined and visualised in context using BED files uploaded to the UCSC genome browser.

**Fig. 1:**
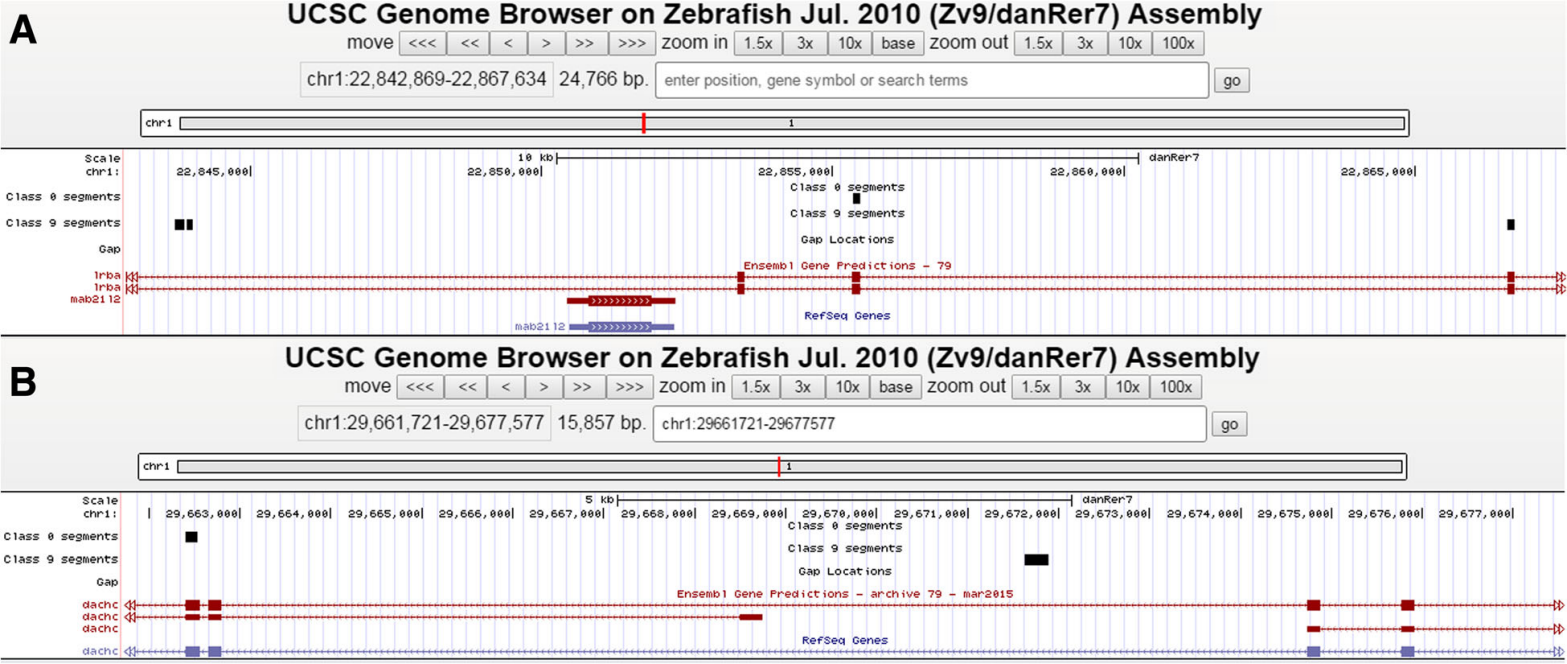
**a** Most conserved segment classes of *lrba* gene. Two BED files uploaded to UCSC genome browser correspond to Class 0 (conservation - 71%) and Class 9 (conservation - 75%) segments of zebrafish chromosome 1. The segments in each of Class 0 and Class 9 overlap annotated exons (wide bars) of *lrba* (ENSDARG00000031108). **b** An intronic region more conserved than exons. The annotated exon (wide bars) of *dachc* (ENSDARG00000003142) coincides with the segment in Class 0. The 261 nt long segment at the right end belongs to Class 9, hence is more conserved than the marked exon

We identified significant variation between genes on the same chromosome in the levels of conservation. We therefore used a gene-specific approach, identifying for each gene the class or classes containing exons and examining these, and more highly conserved classes, for intronic elements. Notably, there are regions within the introns that show equally distinct boundaries and probabilities of belonging to the highly conserved classes as exons, and some intronic regions that are more conserved than coding regions (Fig. 1).

### Conserved intronic elements are widespread in the human, mouse, and zebrafish genomes

Some of the intronic conservation blocks identified were very short, or their assignment to the highly conserved class had a low probability. Therefore, we filtered the results for intronic segments of at least 100 nt in length, such that each position in the region had ≥0.9 probability of belonging to the highly conserved class/classes of each gene in question. Regions that passed this filtering were referred to as putative functional elements (PFEs).

**Fig. 2:**
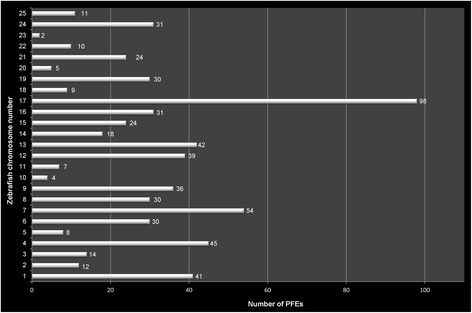
Number of intronic PFEs identified in each zebrafish chromosome. 655 intronic PFEs were identified in 25 zebrafish chromosomes in total. The highest number of PFEs (98) was detected in zebrafish chromosome 17. 34 PFEs were identified in *foxp2* (ENSDARG00000005453) in chromosome 4 and this is the highest number of PFEs found in a single gene followed by 28 PFEs in *npas3* (ENSDARG00000079182 – chromosome 17)

We identified 655 PFEs distributed among 193 zebrafish genes with a median length of 168 nt and with 33% of the PFEs longer than 200 nt (Additional file 1: Table S1). Where the zebrafish genome contained multiple homologues for the human gene we frequently observed the conservation of the PFE in multiple zebrafish genes with 47 PFEs located in zebrafish paralogues corresponding to 23 PFEs in human. All other PFEs were in one-to-one correspondence between zebrafish and human. PFEs were found throughout the genome (Fig. 2), but were not evenly distributed, with 20 genes containing 5–9 PFEs, 17 genes containing 10 or more, and 34 PFEs identified in *foxp2* (ENSDARG00000005453) alone.

### Identified elements correspond to novel, predicted, and known functional sequences

**Fig. 3:**
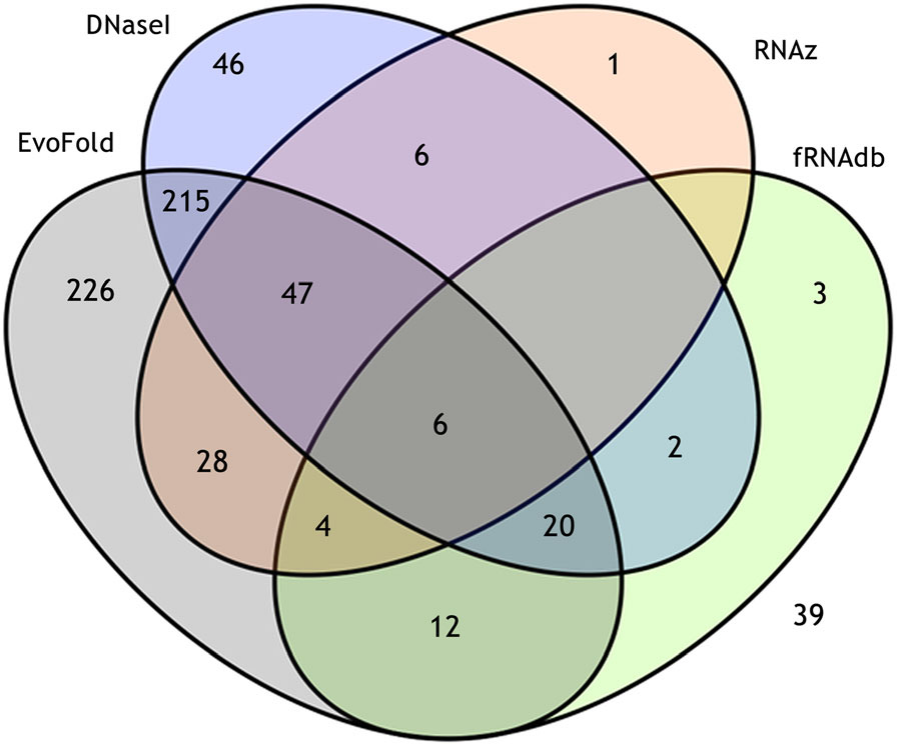
Venn diagram showing the number of genome-wide intronic PFEs supported by other methods. 94% of the PFEs found in the genome-wide analysis overlapped with the functional elements (predicted or experimentally validated) identified in 4 other databases, EvoFold, fRNAdb, RNAz and DNase I footprints. Most of the PFEs overlapped with entries in EvoFold and there were 47 matches with experimentally identified ncRNA transcripts in fRNAdb

To determine if PFEs represent functional elements, and to compare our results to those incorporating secondary structure, we compared PFEs with regions identified by EvoFold, RNAz, DNase I footprinting, and to entries in the functional RNA database. Of the 655 PFEs, 616 (94%) were also identified by other methods (Fig. 3). Note that all of these methods except DNase I footprinting are suggestive of function at the RNA level. In contrast DNase I footprinting suggests the presence of regulatory element binding sites. If we exclude DNase I footprinting, 570 (87%) intronic PFEs have existing annotations suggestive of RNA-level function. EvoFold shared the greatest overlap with changept, 558 PFEs (85%) overlapping with EvoFold predictions, including 174 PFEs containing multiple EvoFold predictions. Only 92 PFEs (15%) were identified by the other predictive tool examined, RNAz (Additional file 2: Table S2).

Comparison to experimental data for DNaseI footprints suggested 342 PFEs (56%) were in protein binding regions. Comparing with fRNAdb, 47 PFEs matched with experimentally identified ncRNA transcripts in the database (Fig. 3 and Additional file 2: Table S2). Of these, 45 mapped to ncRNAs identified in an analysis of the mouse transcriptome [29, 30]. The remaining 2 PFEs were contained in human ncRNA transcripts [31]. Except for one of the human ncRNA transcripts (fRNAdb reference FR407542/FR407474), all other transcripts were substantially longer than the PFEs they matched. This suggests that regions identified as PFEs represent functional domains within longer RNA transcripts.

As an added check to determine if PFEs correspond to ncRNAs, we compared the locations of PFEs with long non-coding RNAs (lncRNAs) identified in zebrafish [32–34]. There were 8 PFEs overlapping with known lncRNAs (Additional file 2: Table S2). Of 655 PFEs, 39 were not identified by the other methods used for comparisons, and thus can be classified as new predictions.

### Characterizing genes containing PFEs

Transcription factors are known to be enriched in intronic ncRNAs [35]. To find if transcription factors were overrepresented in the 193 PFE containing genes we compared the proportion in these genes to that in the genome wide alignments. Results indicated that 40.9% of genes with PFEs (79/193) are transcription factors and 4.7% (9/193) are transcription co-factors (Additional file 3: Table S3) compared to 10.6% (1733/16296) and 1.5% (240/16296) respectively in the genome wide alignment. Therefore PFEs are highly enriched in transcription factors (*p*-value: 1.2e-56, Z-test for comparing proportions).

As an additional analysis, we examined the distribution of Gene Ontology (GO) terms (http://geneontology.org [36]) in the 193 genes with PFEs. GO terms associated with transcription factors (eg: sequence-specific DNA binding transcription factor activity, sequence-specific DNA binding RNA polymerase II transcription factor activity, regulation of transcription DNA-templated, transcription from RNA polymerase II promoter, nucleic acid-templated transcription) were significantly overrepresented in genes containing PFEs (Additional file 4: Table S4).

### Identification of intergenic PFEs

In the genome-wide analysis we also identified 352 intergenic regions that satisfy the PFE selection criteria. Of these, 340 intergenic PFEs (97%) were found to overlap with regions identified by other methods (EvoFold, RNAz, DNase I footprints, or fRNAdb entries, Additional file 5: Table S5). This includes 12 intergenic PFEs that were in ncRNA transcripts according to fRNAdb entries and 11 intergenic PFEs that overlapped with intergenic lncRNAs identified in Pauli et al. 2012. There were 12 highly conserved intergenic regions only identified by program changept.

### Examination of non-coding sequences in a specific pathway

The second part of our study was a pathway-focussed analysis allowing optimisation of the sequence alignment for each gene, and the potential to identify common elements within a pathway. Pathway-focussed analysis was performed on 11 genes encoding transcription factors known to play important roles in myogenesis, and 13 genes encoding muscle proteins. For each gene, human-referenced 3-way alignments were generated independently using LAGAN alignment tool [37].

### Identification of putative functional elements (PFEs)

**Fig. 4:**
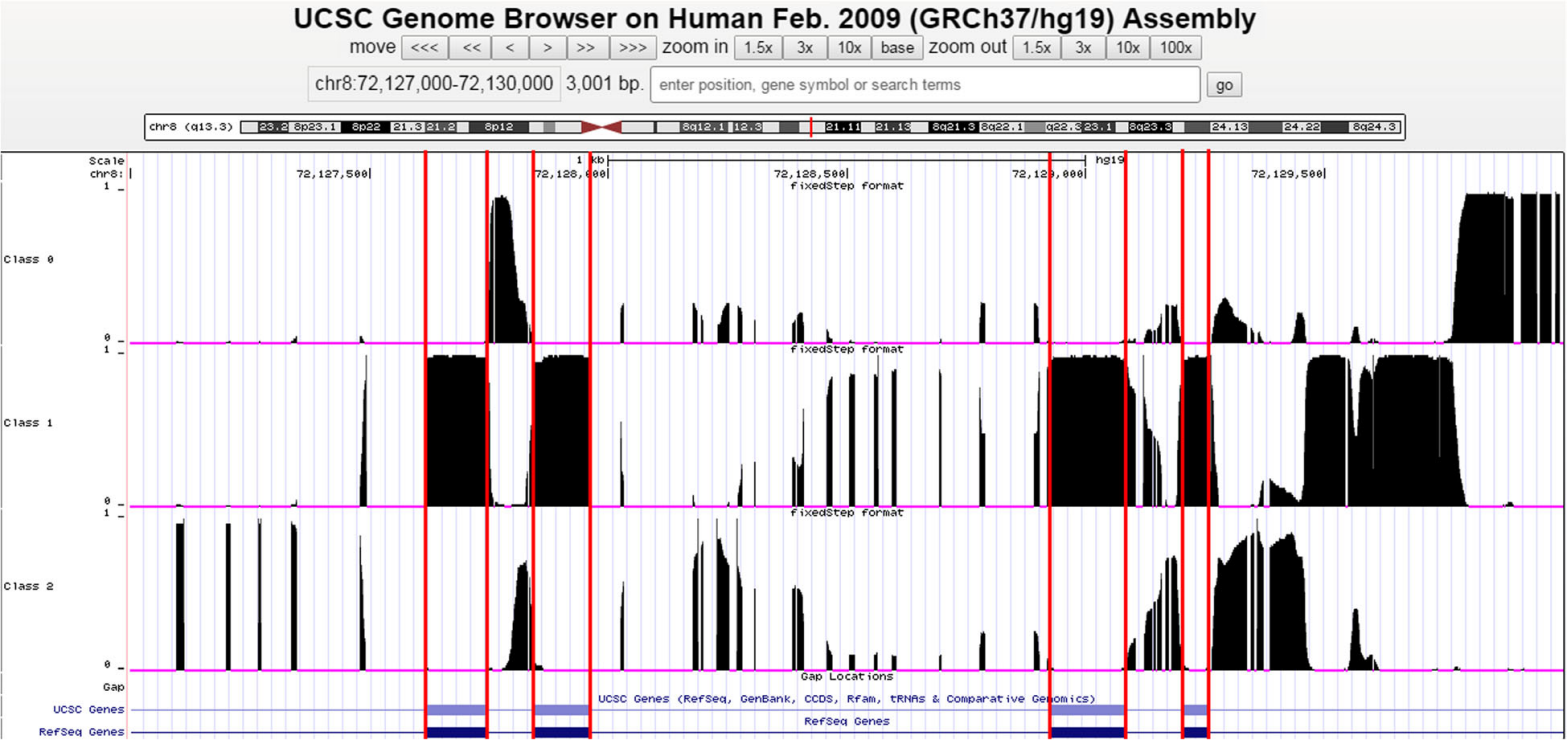
WIG profile of the *eya1*. The top three profiles show, for each sequence position in the human *eya1* DNA sequence (UCSC genomic coordinates chr8: 72,127,000 - 72,130,000), the probability that any base at that position belongs to Class 0 (50% conservation), Class 1 (65% conservation), Class 2 (45% conservation) respectively. At any position, the sum of the three profiles is 1. The two rows below the Class 2 profile display the exons (wide bars) and the introns (thin lines) of *eya1* recorded in the UCSC and RefSeq collections respectively. Exon boundaries are indicated with r*ed vertical lines*. Class 1 corresponds mainly to the mapped exons of *eya1*, and covers regions of high conservation between human, mouse and zebrafish

To search for the most conserved elements in each gene we applied changept to the 3-way alignments corresponding to each of the 24 genes. The profiles were visualised in context using WIG files uploaded to the UCSC genome browser. Fig. 4 demonstrates the effectiveness with which the distinct boundaries of functional elements can be identified. Class 1 is the most conserved class, and sharp changes (from low to high probabilities) in the WIG profile for Class 1 coincide closely with the annotated positions of exons. Intronic regions, not previously reported as functional, are confidently predicted as belonging to the same conservation class that includes all the other exons. These regions were considered for PFE analysis using the same criteria used in the genome-wide analysis (segment length ≥100 nt; profile ≥ 0.9).

We identified 27 PFEs in total, all in introns of 7 of the transcription factors with the majority distributed among *eya1*, *pax3a* and *pax7* (Additional file 6: Table S6). In contrast, no PFEs were identified in the other muscle genes examined. Of the 27 PFEs, only 5 (3 of *pax3a* and 2 of *eya1* PFEs) were identified in our genome-wide analysis suggesting the optimised alignments had a significant impact on the ability to detect PFEs. The median length of PFEs was 222 nt (based on zebrafish sequences) with 15/27 longer than 200 nt, suggesting the length of the elements detected may also be affected by the alignments.

### Comparing PFEs with other supporting evidence

**Fig. 5:**
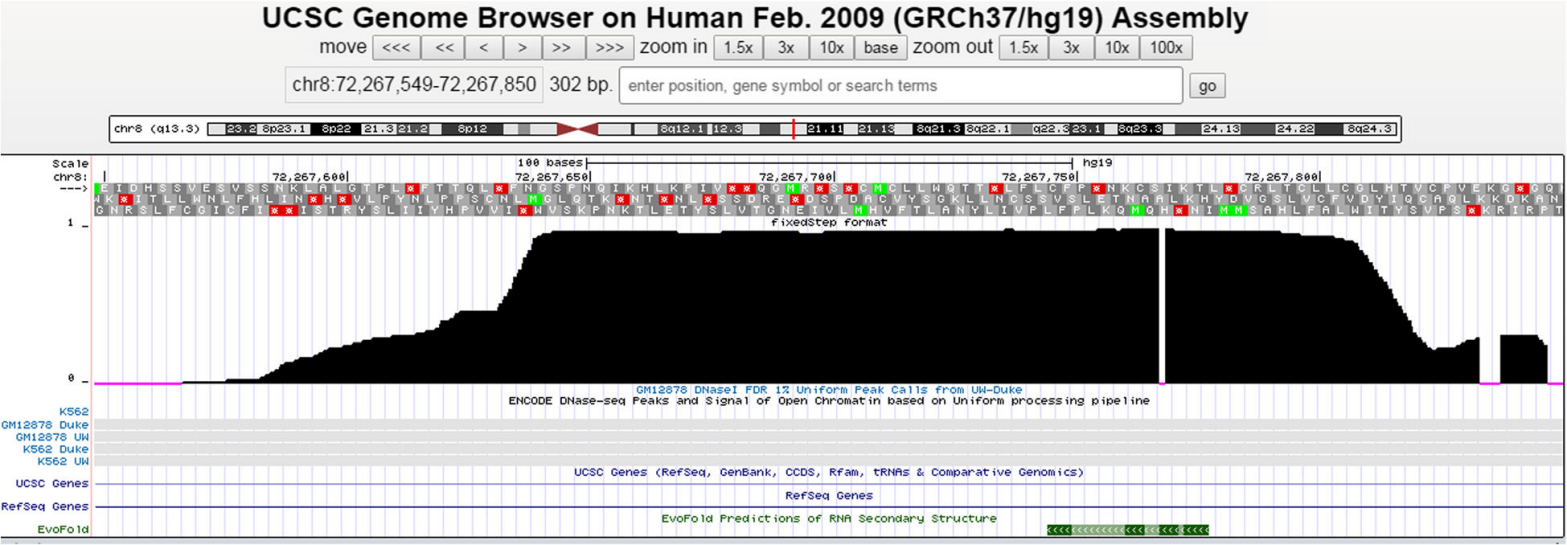
WIG profile of *eya1* PFE 4. This PFE is located within intron 2 of human *eya1* (UCSC genomic coordinates chr8: 72,267,549 -72,267,850). The third bar from the top contains single letter amino acid codes corresponding to the actual protein translation phase. At the bottom, the light grey bar indicates a DNase-seq peak track and the green bar shows that there is an EvoFold prediction within the PFE which also suggest that this region is functional

We analysed the pathway-focussed PFEs using the same methods used in the genome-wide analysis (EvoFold, RNAz, DNase I footprints, and fRNAdb entries). An example WIG profile of a 169 nt long PFE identified in the 3-way alignment of *eya1* is shown in Fig. 5. Three possible translation phases (top) indicate a lack of open reading frame within the region. The overlap of the PFE with a sequence protected in DNA footprinting assays indicates protein binding in this region. Furthermore, the PFE is also predicted by EvoFold.

**Fig. 6:**
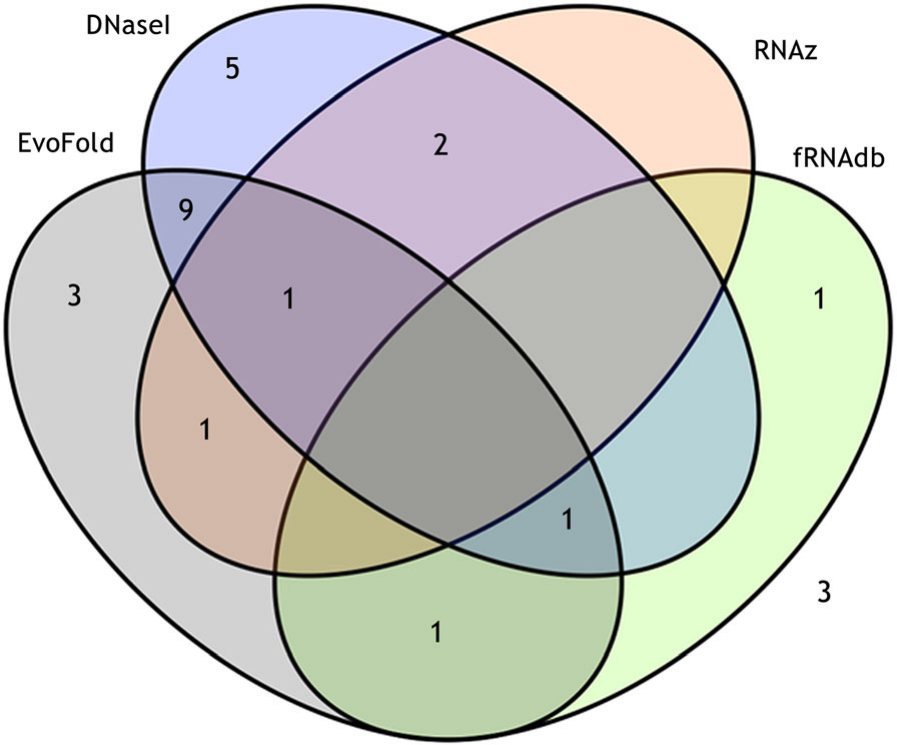
Venn diagram showing the number of pathway-focussed PFEs supported by other methods. 88% of the PFEs found in the pathway-focussed analysis overlapped with the functional elements (predicted or experimentally validated) identified in 4 other databases, EvoFold, fRNAdb, RNAz and DNase I footprints. Most of the PFEs overlapped with entries in either EvoFold or DNase I footprints and there were 3 matches with experimentally identified ncRNA transcripts in fRNAdb

The Venn diagram in Fig. 6 depicts the number of PFEs supported by other evidence and summarised in Table 1 (full details in Additional file 7: Table S7). Of 27 PFEs, 24 were also identified by other methods. (This number reduces to 19 (70%) if DNase I footprinting is not considered.) Out of those 24, the majority of PFEs were identified by either EvoFold (67%) or DNase I footprint regions (75%). Three PFEs overlapped with multiple EvoFold regions (PFE #1 of *pax7b*, #3 and #4 of *pax3a*). In all cases where PFEs overlap with EvoFold regions, the PFEs are longer; this suggests that our analysis has identified extended functional regions.

**Table 1:** Pathway-focussed results: Number of PFEs supported by other methods suggestive of function

Gene	No. of PFEs identified	No. of PFEs contained			
		EvoFold	DNase I footprints	RNAz	ncRNA transcripts (fRNAdb)
*eya1*	6	5	6	0	1
*eya4*	2	1	1	0	0
*pax3*(ZF*a*)^a^	7	5	4	0	1
*pax3*(ZF*b*)	2	1	1	0	1
*pax7*(ZF*b*)	6	4	3	3	0
*shh*(ZF*a*)	2	0	1	0	0
*myf5*	1	0	1	1	0
*six4.3*	1	0	1	0	0
Total	27	16	18	4	3

^a^Note human and mouse DNA sequences of *pax3* are aligned with zebrafish paralog a. Similarly, corresponding zebrafish paralog is mentioned within brackets for other genes if any

Three PFEs matched with two experimentally identified ncRNA transcripts in mouse (Table 2). Both transcripts that mapped to the corresponding region in the mouse genome were substantially longer than the PFEs that they matched. This is consistent with our earlier observation that regions identified as PFEs in the genome-wide analysis, where they overlap with known ncRNAs, are typically shorter than those ncRNAs, and thus may represent functional domains within longer RNA transcripts. The remaining 3 PFEs (PFE 2 of *shha*, PFE 1 of *pax3a* and PFE 6 of *pax7b*) were not identified by any of the 4 other methods used.

**Table 2:** Pathway-focussed results: PFEs matching with experimentally identified ncRNAs in fRNAdb

Gene	UCSC coordinates of human DNA	PFE length (nt)	fRNAdb reference	Length of mapped mouse transcript (nt)
*eya1*	chr8:72,267,639 - 72,267,809	169	FR127136	3697
*pax3(ZFa)*	chr2:223,153,695 - 223,153,821	126	FR205645	1521
*pax3(ZFb)*	chr2:223,153,529 - 223,153,656	113	FR205645	1521

One of the reasons for performing a pathway-focussed analysis was to investigate whether genes in the same pathway contain PFEs with matching sequences. However, we did not find any such matches amongst the 27 PFEs identified in our pathway-focussed analysis.

### Comparing PFEs with CNSs

Another recent list of conserved non-coding sequences (CNSs) was published by Babarinde and Saitou [17]. This list is based on a comparison of mammals using BLASTN. Of the 655 intronic PFEs identified by our criteria, only 195 overlap with these CNSs. However, of the 352 intergenic PFEs we identified, 324 overlapped with CNSs.

### Intronic PFE sequences are expressed in the zebrafish

**Fig. 7:**
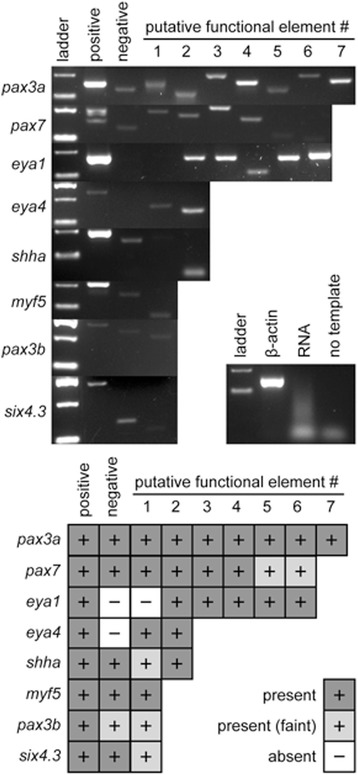
RT-PCR of intronic putative functional elements (PFEs) showing their presence or absence in 24 hpf zebrafish cDNA pools. Each gene has between 1 and 7 PFEs. Exon lane contains an exonic region, spanning an intron, of the gene of interest. Intron lane represents a randomly selected intronic region that was not identified as a PFE. Primers were designed to amplify products with sizes ranging 57-274 bp. The ladder bands shown are 100, 200 and 300 bp. The gels with the two bands of the ladder showing are the 100 and 200 bp bands. The panel insert is a cDNA control. β-actin (exonic spanning an intron) and RNA (RNA used as a template) lanes demonstrate there is no genomic contamination. No template lane rules out contamination of other PCR reagents

To investigate whether the intronic PFEs identified are transcribed, RT-PCR analysis was performed using RNA extracted from 24 hours post-fertilisation (hpf) zebrafish embryos (Fig. 7). Reverse transcription was carried out with a polydT primer to restrict amplification to mature, polyadenylated, mRNA and exclude pre-mRNA. 96% (25/26) of the PFEs showed a positive PCR result indicating transcription of the PFE region (it was not possible to design primers for pax3b PFE2). The positive control in each case confirmed that the gene of interest, from which the intronic PFE is derived, is also expressed at 24hpf. Intronic regions within the gene of interest that were not identified as PFEs were used as controls. The expected result was that there would be no PCR product as is seen for *eya1* and *eya4*. Contrary to expectations, six of the other intronic regions showed a positive PCR result indicating that these intronic regions are also being transcribed. This supports the suggestion that PFEs may be regions within larger transcripts.

**Fig. 8:**
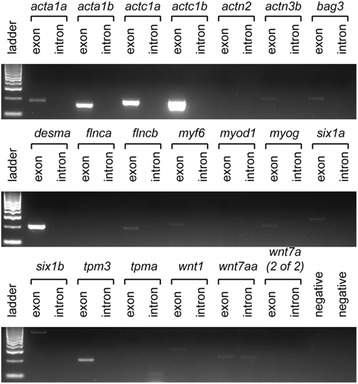
RT-PCR of muscle expressed genes not containing PFEs*.* Exonic sequence amplification is evident for 15 of the genes but only 1 (*wnt7aa*) has amplification of an intronic sequence. Primers were designed to amplify products with sizes ranging 100-638 bp. Lane 1 for each gel contains a 100 bp ladder. The negative lanes are no template controls to rule out genomic DNA contamination

Given the detection of intronic transcripts for 6 out of 8 of the PFE containing genes we wanted to determine if intronic transcripts were found more frequently in PFE containing genes. We examined the expression of 20 additional muscle genes via RT-PCR (Fig. 8). Fifteen of the 20 genes were expressed at the stage examined and for only one of these, *wnt7aa*, was expression of an intronic sequence detectable.

## Discussion

One clue to the possible functions of PFEs is their prevalence in the introns of transcription factors. This was strikingly demonstrated by the pathway-focussed analysis: all PFEs were found in introns of transcription factors, and none in other muscle proteins. Genome-wide, 49.6% of the genes containing PFEs are transcription factors (*p*-value: 1.2e-56, Z-test for comparing proportions). PFEs are found in genes that are not transcription factors, but given that the defining criteria for PFEs are based only on conservation level and length, a mixture of functional types is expected.

PFEs found in the introns of transcription factors could contribute to regulatory interactions in various ways, including: containing binding sites for other transcription factors, containing auto-regulatory binding sites, folding into ncRNAs that interact or form complexes with the host gene, or folding into ncRNAs that interact or form complexes with other genes in a manner that coordinates their expression levels and activity with that of the containing gene.

Our RT-PCR results showed that PFEs from the introns of muscle-related genes are expressed and suggest that they may play a functional role at the RNA level. The identification of the expression of non-PFE sequences also suggests the PFEs are elements within larger intronic transcripts rather than defining the boundary of an intronic ncRNA element. This is supported by the 47 PFEs that matched experimentally verified ncRNAs in human and mouse: all but one of these were from ncRNAs substantially longer than the PFE.

One surprising finding is that only 5 of the 27 PFEs identified in the pathway-focussed analysis were found in the genome-wide analysis. We attribute this to the superior quality of the alignments used in the pathway-focussed analysis, due not only to the use of LAGAN, but also to manual interventions to improve alignment quality. This suggests that the genome-wide analysis may be finding only a fraction of the intronic elements conserved between human and zebrafish, and that improving the quality of genome wide alignments would greatly enhance available methods to detect functional non-coding sequences.

To determine if PFEs correspond to ncRNAs or other regulatory sequences, we compared them to other bioinformatics resources (EvoFold, RNAz, DNase-seq footprints and fRNAdb entries). The majority (85%) of our PFEs identified in the genome-wide study contain EvoFold predicted regions. EvoFold has identified 1445 intronic regions longer than 100 nt in the human genome with the potential to form RNA structures. However a large number of these regions were absent from the alignment we used. This could be due in part to using different alignments with different assemblies and even different species. Our analysis was performed using a more recent alignment including the human 2009 assembly, whereas EvoFold findings are based on an earlier 8-way alignment including the human 2004 assembly. The alignments contain only 4 species in common: human, mouse, zebrafish and fugu. On the other hand, we failed to detect 559 EvoFold predictions that were present in our alignment. This could be due to: (1) failing to satisfy the PFE gap criteria (we rejected segments with a gap of ≥ 20 alignment columns or if the total length of gaps within the segment was ≥10% the length of the segment); or (2) the segments may not be as highly conserved as exons.

This situation was reversed in the pathway-focussed analysis, where we identified 27 PFEs and EvoFold only found 4 regions ≥ 100 nt in the same human genes. This could be attributed to the success of our Bayesian method applied to an improved alignment used in the pathway-focussed analysis.

Ninety-seven (15%) of the PFEs identified in the genome-wide analysis do not contain EvoFold regions and are not within 30 nt of an EvoFold region. Of these, 61% (59) overlap with either RNAz, DNase I footprints, or fRNAdb entries. Moreover, 11 PFEs identified in the pathway-focussed analysis do not contain EvoFold predictions but were all found to be expressed in our RT-PCR results. In addition to identifying putative ncRNAs not identified by EvoFold, our method typically extends the length of the predicted functional regions, so much so that many of our PFEs contain two or more EvoFold predictions. In particular, in the pathway-focussed results, PFEs that contain an EvoFold prediction are substantially longer than that Evofold prediction.

The intronic PFEs we have identified differ substantially from the CNSs of Babarinde and Saitou [17], with approximately 70% of intronic PFEs not overlapping CNSs. In contrast, almost all of our intergenic PFEs overlap with CNSs. One reason for differences between PFEs and CNSs is that they are based on different species comparisons: human, mouse and zebrafish in the former case and human, mouse, dog, cattle and chicken in the latter. However, the novel intronic PFEs we detected may be due at least in part to our Bayesian change-point methodology, which uses information about sequence composition and mutation frequency in addition to conservation to identify segmental structure. Another distinctive feature of our methodology is that the criteria for identifying PFEs depends on the local characteristics of the sequence. In particular, we identify which segment classes contain the exons of the containing gene, and extract PFEs from these classes and more highly conserved classes. This may explain why our method identified many novel PFEs in introns, where the conservation level of the adjacent exons provides a benchmark for the local level of similarity of conserved sequences.

## Conclusions

Our study provides a systematic process centred on a Bayesian segmentation method to identify putative intronic functional elements in genomes that may contain ncRNAs and other regulatory sequences. We carried out independent genome-wide and pathway-focussed analyses identifying conserved non-coding sequences that we termed Putative Functional Elements (PFEs) in human, mouse and zebrafish. Comparison of PFEs to other databases indicative of non-protein-coding function revealed further evidence of function for most of our PFEs, with many of our PFEs substantially increasing the sequence length of other predictions. PFEs identified in our pathway-focussed analyses were shown to be expressed in 24hpf zebrafish embryos, with evidence that expressed elements are longer even than our PFEs, suggesting that computational methods of detecting functional elements, including our own, are finding conserved domains within longer elements of currently unknown extent. PFEs are significantly enriched in the introns of transcription factors, suggesting many of them play roles in the regulatory networks of the containing TF.

## Methods

### Genome-wide PFE analysis

Multiz 8-way alignment was downloaded from UCSC genome browser (http://hgdownload.soe.ucsc.edu/goldenPath/danRer7/multiz8way/). The assemblies used in the alignments were: zebrafish: Zv9/ danRer7; human: hg19/GRCh37 and mouse: GRCM38/ mm9. For each zebrafish chromosome, the 3-way alignment (zebrafish-referenced) was extracted using program mafExtractor (https://github.com/dentearl/mafTools/tree/master/mafExtractor) giving 25 alignments in total, one for each zebrafish chromosome.

### Pathway-focussed PFE analysis

Transcription factors of the myogenesis pathway: *eya1, eya4, pax3, pax7, six4.3, myf5, shh, six1, myod1, myog, myf6* and other muscle expressed proteins: *wnt1, wnt7a, acta1, actc1, actn2, actn3, bag3, des, flnc, tpm3, myh7, tnnt1, nebulin* were analysed. Human, mouse and zebrafish DNA sequences for each of 24 genes were downloaded from Ensembl genome browser (http://www.ensembl.org/index.html; zebrafish: Zv9; human: GRCh37 and mouse: NCBIM37). For 10 of these 24 genes (*pax3, shh, six1, wnt7a, acta, actc, actn3, desm, flnc, tpm3*), there are 2 paralogues in zebrafish and for *myh7* there are 3 paralogues. Thus a separate 3-way alignment was generated for each of these, giving a total of 36 alignments (For *pax7*, only *pax7b* was used as we couldn’t identify the complete sequence of *pax7a*). We used LAGAN [37] to perform the 3-way alignments (human-referenced) using default parameters. For two cases where we noticed mis-alignments of exons (*myf6, wnt7aa*), those sequences were aligned separately using ClustalW2 (http://www.ebi.ac.uk/Tools/msa/clustalw2/) effectively forcing exons to align. We then combined the ClustalW2 results (partial alignments) with the original LAGAN alignments. For example, we performed the following steps to align the sequences of *myf6*: 1. We obtained the 3-way LAGAN alignment of *myf6* using 3 FASTA files containing human, mouse and zebrafish DNA sequences. 2. We inspected the 3-way alignment to determine whether exons of *myf6* were correctly aligned. Here we noticed that zebrafish exon 2 was not aligned to the corresponding exons in human or mouse. 3. We provided the exon 2 sequences of the three species to ClustalW2 to align separately. 4. We replaced *myf6* zebrafish exon 2 sequence with the human exon 2 sequence in the original zebrafish FASTA file. 5. We used LAGAN to realign the human and mouse *myf6* sequences with the modified zebrafish *myf6* sequence. LAGAN aligned all copies of exon 2. 6. Finally, we replaced the exon 2 aligned section of the new 3-way alignment file (output obtained from step 5) with the alignment of exon 2 obtained using the ClustalW2 program (output obtained from step 3).

### Transformation of alignments

Each of the 3-way alignments was transformed into a single 32-character sequence ($A=\left\{ a, b, c, d, e, f, g, h, i, j, k, l, m, n, o, p, q, r, s, t, u, v, w, x, y, z, U, V, W, X, Y, Z\right\}\Big)$ using the following encoding. This sequence was used as the input for program changept. Alignment columns with complementary bases were also encoded using the same characters: for example, an alignment column containing G, A and T for zebrafish, mouse and human respectively would be encoded using the same character as an alignment column containing the equivalent complementary bases C, T and A, namely n. Thus the coding of an alignment is the same regardless of the strand analysed.

- Zebrafish: ACGTACGTACGTACGTACGTACGTACGTACGT
- Mouse: AAAACCCCGGGGTTTTAAAACCCCGGGGTTTT
- Human: AAAAAAAAAAAAAAAACCCCCCCCCCCCCCCC
- Symbol: abcdefghijklmnopqrstuvwxyzUVWXYZ

The insertions and deletions in the alignment were excluded from analysis. In the genome-wide analysis, discontinuous alignment blocks with respect to each species were also separated by using a ‘#’ symbol. The ‘#’ symbol is considered as a fixed change-point in the model.

Occasionally changept identified only one class of segments in segmenting the 3-way alignments of relatively short genes (for example *shh, myog, six1, six4.3* in pathway-focussed analysis). This problem was overcome by concatenating the 32-character sequences of such genes, thus providing changept a larger sample to segment.

### Change-point analysis

A full description of the change-point model can be found in previous papers [26, 27, 38]. In summary, the sequences generated for 3-way alignments for each of the genes/chromosomes were separately run through changept to find positions (change-points) in the sequences that delineate homogeneous segments. Character frequencies within each segment are modelled as a multinomial distribution with parameter$\theta =\left({\theta}_a,{\theta}_b,\dots, {\theta}_Y,{\theta}_Z\right)$, where $\theta$ is drawn from one of *T* Dirichlet distributions. As the number of classes (*T*) is unknown *a priori*, independent runs with different numbers of classes were performed. The generalized Gibbs sampler [38] was used to sample from the varying dimensional space: it allows the number of change-points to vary. Each model was run with varying values of *T* for 1,000 iterations. Information criteria were then used to select the value of *T*.

### Assessing convergence

The convergence of the model was assessed by plotting the log-likelihood of each of the 1000 iterations. The *burn-in* phase is characterised by an upward trend in the log-likelihood.

### Model selection

To determine the optimal number of classes for each alignment, we calculated approximations to three information criterion values- Akaike Information Criterion (AIC), Bayesian Information Criterion (BIC), and Deviance Information Criterion (DICV) - using post burn-in samples (Additional file 8: Figure S1). These approximations are discussed in [39]. The model with the smallest information criterion value was considered optimal. However, model selection was not purely based on this method. A subjective judgement was made on which model to choose by investigating the mixture proportions; a model containing classes with very low mixture proportions was considered to be an over-fitted model and thus a model with a smaller number of classes was selected. In combination with this method, we also used an alternative model selection method, by investigating the stability of segment classes [28]. Stability of classes was assessed based on time-series plots of conservation levels versus sample number. Classes which were highly variable in conservation levels were deemed unstable (Additional file 9: Figure S2). The number of segment classes selected for each zebrafish chromosome, and the conservation level and GC content of each class, is listed in Additional file 10: Table S8.

### Quantifying the conservation level of segment classes

Changept employs Markov Chain Monte Carlo sampling. The individual character frequencies within each class were calculated at each iteration. To determine the conservation level of each class for the selected model, the mean proportion of alignment matches ($E\left(\theta \right)$) was calculated for each iteration of the sampler.

$$ E\left(\theta \right)=\frac{\theta_a+{\theta}_v}{{\displaystyle {\sum}_{j\in A}}{\theta}_j} $$

Here characters ‘$a$’ and ‘$v$’ represent conserved bases. These values were plotted against each iteration number (Additional file 9: Figure S2). These conservation plots were also used to identify the ‘burn-in’ period as a second method. For example, Additional file 9: Figure S2(A) shows that convergence to the limiting distribution has occurred rapidly, apparently within the first 50 iterations.

### Assignment of sequences to classes

We used the *readcp* program (part of the changept package) to calculate profile values showing the probability that each sequence position belongs to a given class of the chosen model. These posterior probabilities are estimated by Monte Carlo integration. A complete description of how changept and *readcp* were applied can be found in [40, 41].

### Identifying putative functional elements

PFEs were identified for the 3-way alignments of each gene using the following criteria: an intronic segment of at least 100 nt in length, such that each position had ≥ 0.9 probability of belonging to the most conserved segment class or classes overlapping that gene. The most conserved class or classes were determined by identifying those classes that overlapped exons, or had higher levels of conservation than classes that overlapped exons. Note this criteria is gene-specific. As changept skips gaps in the alignment, gaps were considered in the following manner: a segment was not considered continuous if there was a gap of ≥ 20 alignment columns or if the total length of gaps within the segment was ≥10% the length of the segment. In the genome-wide analysis, regions that satisfy PFE criteria belonging to the most conserved class of the selected model corresponding to each zebrafish chromosome, but not located in genic regions were referred as ‘intergenic PFEs’. PFEs predicted in alignments between non-homologous genes were discarded (10 PFEs located in 7 alignments, Additional file 11: Table S9).

### Creation of wiggle tracks and BED files

The *readcp* output was used to generate BED files or wiggle tracks (one for each class in the final model) so that results could be plotted as a profile alongside gene tracks and other information in the UCSC browser.

In the genome-wide analysis we used the more compact BED file format to handle the large amount of data. The positions of segments matching PFE criterion (minimum segment length of 100 nt with profile ≥ 0.9 and same gap criterion as above) in each class and in each model were recorded in BED format with genomic coordinates relative to zebrafish. We used ‘intersect’ BEDtool (http://bedtools.readthedocs.org/en/latest/content/tools/intersect.html) to find the segment class (or classes) that overlap with annotated exons (3’ untranslated region (UTR) exons, 5’ UTR exons and the coding exons downloaded from UCSC table browser) of the gene in question. Sometimes there was more than one class corresponding to annotated exons of the gene (Fig. 1) and occasionally segments satisfying PFE criteria were found to be located in a class more highly conserved than a class corresponding to marked exons (for example, there is a PFE in Class 9 in Fig. 1b). Thus in each gene, segments that were conserved at a level comparable or higher than exons were considered for PFE analysis. In our analysis we only reported PFEs with conservation level > 50%.

Wiggle tracks were used in the pathway-focussed analysis. The WIG profile for a selected class shows the probability that the base at a particular position in the sequence belongs to the class in question, thus every position has an associated value between 0 and 1 (Fig. 4). In this analysis, we examined the wiggle track of the most conserved segment class (for example, Class 1 of Fig. 4).

### Comparison to alternative methods for identifying functional non-coding sequences

EvoFold: Human genomic coordinates of EvoFold regions were downloaded in BED format using UCSC table browser. To check the overlap between PFEs and EvoFold regions, we used BEDtool -intersect.

DNase I footprints: we used the database of DNase-seq footprints identified by the ENCODE project [42] in their large-scale analysis of 41 different human cell types. The data (combined.fps.gz) was downloaded from link ftp://ftp.ebi.ac.uk/pub/databases/ensembl/encode/integration_data_jan2011/byDataType/footprints/jan2011/. Once again BEDtool -intersect was used to check the overlap between PFEs and DNase-seq footprints.

*fRNAdb*: The ‘BLAST’ function of fRNAdb database [43] was used to search for fRNAdb entries (ncRNA transcripts and RNAz regions) with high sequence similarity to human sequences of each PFE identified in our analysis.

### Zebrafish maintenance and cDNA synthesis

Zebrafish were maintained as previously described [44]. RNA was collected from 24hpf wild-type embryos using TRI-Reagent® (Sigma-Aldrich) and treated with DNAse (Promega) to remove genomic DNA. cDNA was synthesised using the ProtoScript® II First Strand cDNA Synthesis Kit (NEB) using polydT primers only to prevent transcription of pre-mRNA prior to removal of introns and polyadenylation.

### Polymerase chain reaction and gel electrophoresis

Reverse transcriptase PCR was performed using GoTaq Green Master Mix (Promega). Samples were amplified for 30 cycles with an annealing temperature of 57 °C. 15 μl of each sample was run on a 3% TBE gel, supplemented with GelRed (Biotium), at 60V for 3 h. Positive control sequences were obtained using Ensembl Genome Browser (http://www.ensembl.org/index.html) and regions spanning introns of the genes of interest were selected. PFE and negative control sequences were obtained after analysis with changept and primers were designed using the online software Primer3 (http://bioinfo.ut.ee/primer3).

### Analysis of GO terms

To examine the proportion of genes containing PFEs that are either transcription factors, transcription co-factors or chromatin remodelling factors, we first downloaded the Ensembl gene list associated with each category. In total, there were 2345 transcription factors, 315 transcription co-factors and 100 chromatin remodelling factors in the database. Next we used BEDtool-intersect to check how many genes were represented in genome-wide 3 way alignments. 16296 genes (from total 32475 Ensembl genes) overlapped with the segments recorded in our BED files. The final step was to examine the proportion of transcription factors, transcription co-factors and chromatin remodelling factors in aligned 16296 genes using 3 corresponding lists downloaded from AnimalTFDB (http://bioinfo.life.hust.edu.cn/AnimalTFDB/index.shtml; Zhang et al. 2012).

To perform GO enrichment analysis, we used ‘AmiGO’ web interface accessible at http://amigo.geneontology.org/amigo [45]. We obtained significant GO terms (with *p*-value <0.05) in each of three sub-ontologies: Biological Process, Molecular Function, and Cellular Component using 193 zebrafish genes containing PFEs. Further, we manually filtered GO terms associated with ‘DNA binding’, ‘regulation of gene expression’, ‘sequence-specific DNA binding’ and ‘nucleic acid binding’ to check if any of the genes in the sample were classified as transcription factors using existing evidence.

## Additional files

Additional file 1: Table S1. UCSC genomic coordinates and zebrafish gene IDs (Ensembl) of intronic PFEs identified in genome-wide analysis. This table provides the location of the PFEs identified in both the zebrafish and human genomes. Where multiple PFEs in zebrafish map to the same location in the human genome these are highlighted in yellow. (XLSX 48 kb)

Additional file 2: Table S2. Supporting evidence for intronic PFEs identified in genome-wide analysis. For each intronic PFE overlap with Evofold or RNAz predictions, DNaseI footprint data, entry in the fRNAdb, or previous lncRNA publications is presented. (XLSX 63 kb)

Additional file 3: Table S3. Genes with PFEs classified as transcription factors. The Ensembl ID for all gene containing PFEs that have been classified as transcription factors or transcription co-factors is provided as identified by AnimalTFDB (http://bioinfo.life.hust.edu.cn/AnimalTFDB/index.shtml; Zhang et al. 2012). Eight extra genes containing PFEs not identified by AnimalTFDB were found to be enriched with GO terms associated transcription factors. (XLSX 10 kb)

Additional file 4: Table S4. GO terms related to Transcription Factors. The frequency of GO terms relating to transcription factors in gene containing PFEs, compared to all zebrafish genomes. (XLSX 10 kb)

Additional file 5: Table S5. Intergenic PFEs identified in genome-wide analysis. For each intergenic PFE overlap with Evofold or RNAz predictions, DNaseI footprint data, entry in the fRNAdb, or previous lncRNA publications is presented. (XLSX 41 kb)

Additional file 6: Table S6. UCSC genomic coordinates of PFEs identified in pathway-focussed analysis. The genomic coordinates in both the zebrafish and human genomes are provided for each of the PFEs identified in the pathway focussed analysis. (XLSX 10 kb)

Additional file 7: Table S7. Supporting evidence for PFEs identified in pathway-focussed analysis. For each PFE identified in the pathway focussed analysis overlap with Evofold or RNAz predictions, DNaseI footprint data, or entry in the fRNAdb is presented (XLSX 11 kb)

Additional file 8: Figure S1. Model selection for *eya1*. Approximations to well-known information criteria AIC, BIC and DICV for 1-12 classes. Generally, a lower value of the information criteria indicates a better model. BIC clearly suggests a 3-class model. The first local minimum of AIC and DICV has also occurred at the 3-class model. Therefore we selected a 3-class model for this data. (TIFF 48 kb)

Additional file 9: Figure S2. Model selection of chromosome 1 alignment. Figure shows the time series plots of conservation level versus iteration number for each class of (A) 19-class model; and (B) 20-class model. In (A), all classes have stable conservation levels and in (B), one of the classes has a widely varying conservation level. Thus the 19-class model was selected for chromosome 1 alignment. Figure (A) also shows that the model has converged rapidly. (TIFF 145 kb)

Additional file 10: Table S8. Optimal number of classes selected for each model of each zebrafish chromosome. The number of segment classes selected for each zebrafish chromosome and the conservation level and GC content of each class. (XLSX 63 kb)

Additional file 11: Table S9. PFEs discarded from the genome-wide analysis. PFEs identified in non-homologous genes in the human and zebrafish genomes, removed from the genome-wide analysis. (XLSX 9 kb)

## Abbreviations

AIC: Akaike information criterion
BIC: Bayesian information criterion
CNS: conserved non-coding sequence
DICV: deviance information criterion
GO: gene ontology
hpf: hours post-fertilisation
ncRNAs: non-coding RNAs
nt: nucleotide
PFE: putative functional element
RT-PCR: reverse transcriptase polymerase chain reaction
TFBS: transcription factor binding site
UTR: untranslated region

## References

[1] Khalil AM, Guttman M, Huarte M, Garber M, Raj A, Rivea Morales D (2009). Many human large intergenic noncoding RNAs associate with chromatin-modifying complexes and affect gene expression. Proc Natl Acad Sci U S A.

[2] Koziol MJ, Rinn JL (2010). RNA traffic control of chromatin complexes. Curr Opin Genet Dev.

[3] Rinn JL, Kertesz M, Wang JK, Squazzo SL, Xu X, Brugmann SA (2007). Functional demarcation of active and silent chromatin domains in human HOX loci by noncoding RNAs. Cell.

[4] Corey DR (2005). Regulating mammalian transcription with RNA. Trends Biochem Sci.

[5] Mattick JS, Makunin IV (2005). Small regulatory RNAs in mammals. Hum Mol Genet.

[6] Kishore S, Stamm S (2006). The snoRNA HBII-52 regulates alternative splicing of the serotonin receptor 2C. Science.

[7] Mattick JS, Makunin IV. Non-coding RNA. Hum Mol Genet. 2006;15 Spec No 1:R17–29. 10.1093/hmg/ddl046 16651366

[8] Storz G, Opdyke JA, Zhang A (2004). Controlling mRNA stability and translation with small, non-coding RNAs. Curr Opin Microbiol.

[9] Zuker M (2003). Mfold web server for nucleic acid folding and hybridization prediction. Nucl Acids Res.

[10] Hofacker IL, Stadler PF (2006). Memory efficient folding algorithms for circular RNA secondary structures. Bioinformatics.

[11] Gruber AR, Findeiß S, Washietl S, Hofacker IL, Stadler PF (2010). RNAz 2.0: Improved noncoding RNA detection. Pac Symp Biocomput.

[12] Pedersen JS, Bejerano G, Siepel A, Rosenbloom K, Lindblad-Toh K, Lander ES (2006). Identification and classification of conserved RNA secondary structures in the human genome. PLoS Comput Biol.

[13] Levy S, Hannenhalli S, Workman C (2001). Enrichment of regulatory signals in conserved non-coding genomic sequence. Bioinformatics.

[14] Bejerano G, Pheasant M, Makunin I, Stephen S, Kent WJ, Mattick JS (2004). Ultraconserved elements in the human genome. Science.

[15] Woolfe A, Goodson M, Goode DK, Snell P, McEwen GK, Vavouri T (2005). Highly conserved non-coding sequences are associated with vertebrate development. PLoS Biol.

[16] Babarinde IA, Saitou N (2013). Heterogeneous tempo and mode of conserved noncoding sequence evolution among four mammalian orders. Genome Biol Evol.

[17] Babarinde IA, Saitou N (2016). Genomic Locations of Conserved Noncoding Sequences and Their Proximal Protein-Coding Genes in Mammalian Expression Dynamics. Mol Biol Evol.

[18] Hemberg M, Gray JM, Cloonan N, Kuersten S, Grimmond S, Greenberg ME (2012). Integrated genome analysis suggests that most conserved non-coding sequences are regulatory factor binding sites. Nucleic Acids Res.

[19] Shen Y, Yue F, McCleary DF, Ye Z, Edsall L, Kuan S (2012). A map of the cis-regulatory sequences in the mouse genome. Nature.

[20] Takahashi M, Saitou N (2012). Identification and characterization of lineage-specific highly conserved noncoding sequences in Mammalian genomes. Genome Biol Evol.

[21] Sandelin A, Bailey P, Bruce S, Engström PG, Klos JM, Wasserman WW (2004). Arrays of ultraconserved non-coding regions span the loci of key developmental genes in vertebrate genomes. BMC Genomics.

[22] Tajima F (1991). Determination of window size for analyzing DNA sequences. J Mol Evol.

[23] Braun JV, Muller H-G (1998). Statistical methods for DNA sequence segmentation. Statist Sci.

[24] Algama M, Keith JM (2014). Investigating genomic structure using *changept*: A Bayesian segmentation model. Comput Struct Biotechnol J.

[25] Oldmeadow C, Mengersen K, Mattick JS, Keith JM (2010). Multiple Evolutionary Rate Classes in Animal Genome Evolution. Mol Biol Evol.

[26] Keith JM (2006). Segmenting eukaryotic genomes with the Generalized Gibbs Sampler. J Comput Biol.

[27] Keith JM, Adams P, Stephen S, Mattick JS (2008). Delineating slowly and rapidly evolving fractions of the Drosophila genome. J Comput Biol.

[28] Algama M, Oldmeadow C, Tasker E, Mengersen K, Keith JM (2014). Drosophila 3' UTRs are more complex than protein-coding sequences. PLoS One.

[29] Okazaki Y, Furuno M, Kasukawa T, Adachi J, Bono H, Kondo S (2002). Analysis of the mouse transcriptome based on functional annotation of 60,770 full-length cDNAs. Nature.

[30] Carninci P, Kasukawa T, Katayama S, Gough J, Frith MC, Maeda N (2005). The transcriptional landscape of the mammalian genome. Science.

[31] Imanishi T, Itoh T, Suzuki Y, O'Donovan C, Fukuchi S, Koyanagi KO (2004). Integrative annotation of 21,037 human genes validated by full-length cDNA clones. PLoS Biol.

[32] Ulitsky I, Shkumatava A, Jan CH, Sive H, Bartel DP (2011). Conserved Function of lincRNAs in Vertebrate Embryonic Development despite Rapid Sequence Evolution. Cell.

[33] Pauli A, Valen E, Lin MF, Garber M, Vastenhouw NL, Levin JZ (2012). Systematic identification of long noncoding RNAs expressed during zebrafish embryogenesis. Genome Res.

[34] Kaushik K, Leonard VE, KV S, Lalwani MK, Jalali S, Patowary A, et al. Dynamic Expression of Long Non-Coding RNAs (lncRNAs) in Adult Zebrafish. Ramchandran R, editor. PLoS ONE. Public Library of Science; 2013;8:e83616. 10.1371/journal.pone.0083616 PMC3877055 24391796

[35] Nakaya HI, Amaral PP, Louro R, Lopes A, Fachel AA, Moreira YB (2007). Genome mapping and expression analyses of human intronic noncoding RNAs reveal tissue-specific patterns and enrichment in genes related to regulation of transcription. Genome Biol.

[36] Consortium TGO (2013). Gene Ontology Annotations and Resources. Nucleic Acids Res.

[37] Brudno M, Do CB, Cooper GM, Kim MF, Davydov E, Program NCS (2003). LAGAN and Multi-LAGAN: efficient tools for large-scale multiple alignment of genomic DNA. Genome Res.

[38] Keith JM, Kroese DP, Bryant D (2004). A Generalized Markov Sampler. Methodol Comput Appl Probab.

[39] Oldmeadow C, Keith JM (2011). Model Selection in Bayesian Segmentation of multiple DNA alignments. Bioinformatics.

[40] Keith JM (2008). Sequence segmentation. Methods Mol Biol.

[41] Tasker E, Keith JM (2017). Sequence Segmentation with changeptGUI. Methods Mol Biol.

[42] Neph S, Vierstra J, Stergachis AB, Reynolds AP, Haugen E, Vernot B (2012). An expansive human regulatory lexicon encoded in transcription factor footprints. Nature.

[43] Kin T, Yamada K, Terai G, Okida H, Yoshinari Y, Ono Y (2007). fRNAdb: a platform for mining/annotating functional RNA candidates from non-coding RNA sequences. Nucl Acids Res.

[44] Westerfield M (2007). The Zebrafish Book.

[45] Carbon S, Ireland I, Mungall CJ, Shu SQ, Marshall B, Lewis S (2008). AmiGO: online access to ontology and annotation data. Bioinformatics.

