# Supplementary figures and images for: Genome-wide identification of conserved intronic non-coding sequences using a Bayesian segmentation approach
Source: BMC Genomics. 2017 Mar 27;18:259. doi: 10.1186/s12864-017-3645-2 (PMC5369223; doi:10.1186/s12864-017-3645-2)

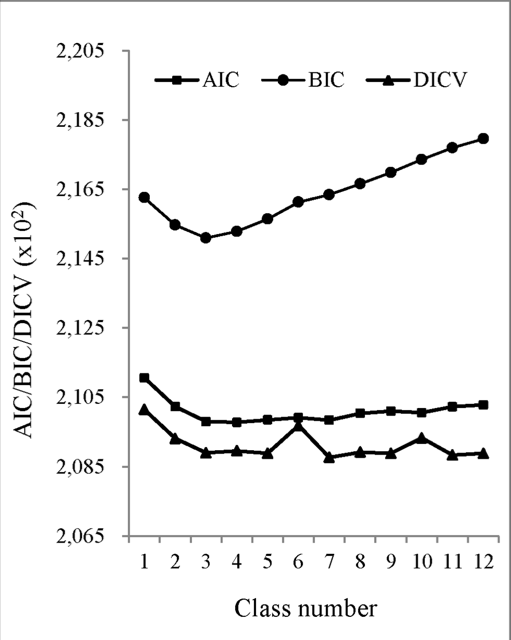

Supplement: Supplementary file 8 — Model selection for eya1. Approximations to well-known information criteria AIC, BIC and DICV for 1-12 classes. Generally, a lower value of the information criteria indicates a better model. BIC clearly suggests a 3-class model. The first local minimum of AIC and DICV has also occurred at the 3-class model. Therefore we selected a 3-class model for this data. (TIFF 48 kb) [file 12864_2017_3645_MOESM8_ESM.tiff]

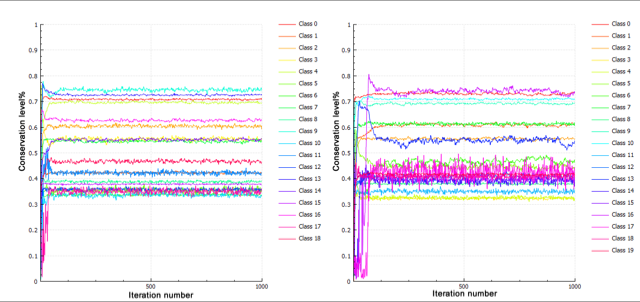

Supplement: Supplementary file 9 — Model selection of chromosome 1 alignment. Figure shows the time series plots of conservation level versus iteration number for each class of (A) 19-class model; and (B) 20-class model. In (A), all classes have stable conservation levels and in (B), one of the classes has a widely varying conservation level. Thus the 19-class model was selected for chromosome 1 alignment. Figure (A) also shows that the model has converged rapidly. (TIFF 145 kb) [file 12864_2017_3645_MOESM9_ESM.tiff]
